# Book Review

**Published:** 1928

**Authors:** 


### Reviews of Books.

**Handbook of Diseases of the Nose, Throat and Ear.** By W. S. Syme, M.D., F.R.F.P. and S.G., F.R.S.E. Second edition. Pp. xvi., 395. Illustrated. Edinburgh : E. & S. Livingstone. 1927. Price 12s. 6d.—In the second edition this book has been enlarged by addition to the number of diagrams and the inclusion of a number of coloured plates and X-ray photographs. The text has also been brought up to date, notably the section on endoscopy. The author's aim has been to present a readable book of reasonable size on the subject, and in this he has succeeded. The book is readable and contains much that is of value, but while the clinical pictures are well set out the question of differential diagnosis receives scant attention. Space devoted to this aspect of the subject would be better utilised than space taken up by detailed description of such operations as laryngo-fissure. In the publishers' desire to economise space the various sections are made continuous, and one has to refer to the table of contents at the beginning of the book to find whether any definite method of arrangement has been adopted. The description in one coloured plate of the left and right tympanic membranes as right and left respectively is prominent among the few printers' errors.

**Diagnosis and Spiritual Healing.** By F. G. Crookshank, M.D. (Lond.), F.R.C.P. Pp. 101. London: Kegan Paul, Trench, Trübner. 1927. Price 2s. 6d.—This book is entirely philosophical. The author deprecates the divorce of medicine, as a science of observed facts, from philosophy. He utters a word of warning against the theory of pure realism in medicine as prejudicial to clarity of thought. A chapter is devoted to the history of attempts to define the word "diagnosis," and he shows the influence of formal logic upon the minds of physicians of former days. The most interesting part of his book is his attempt to show that difference in diagnostic method is mainly related to difference in psychological types of observers. He finds in the rival schools of Coans and Cnidians, as presented in the teaching of Hippocrates and Galen, the prototypes of the empirist and rationalistic methods. The former natural and descriptive, the latter conventional and dogmatic. The Cnidians overstrain diagnosis, reducing it to a system that divides and sub-divides diseases into endless varieties or species.

Their aim was not to assay the patient's state, but to identify his malady with a standardised ideal. In modern medicine these early types correspond to the clinician, as opposed to the statistician and laboratory worker. The difference is less one of doctrine than of method. The second section of the book is a philosophic discussion of the attitude adopted by theologians and the profession as a whole towards spiritual healing. The author maintains that so long as the medical profession accepts the materialistic dualism of mind and matter they cannot adequately deal with the facts of experience, and can never appreciate the effect of mind on organic disease. Such an attitude is inconsistent with any belief in Divine interference in the relatively settled order of Nature. He also asks the Church, more particularly the Protestant Church, to define their attitude with relation to mind and matter. He shows that the antithesis between functional and organic disease is absurd. He says, "We had better be frank and admit that the distinction was invented in order that we might say that organic disease is what we say we can cure, but don't, while functional disease is what the quacks cure, and we wish to goodness we could." The author says that, in virtue of his own dispassionate attitude towards the question of the mind-matter relation, he is prepared to examine experience in every case on its own merit. He believes in the instinctive or intuitive powers of certain unofficial healers, and he holds that spiritual healing (psychotherapy) in its higher manifestations is frequently the result of religious experience. Many of the sufferers are subject to inhibitions, the result of mental conflict: the solution of such conflict in the hands of the spiritual healer is the potent factor. He regards the scope of psychotherapy as coterminous with disease, but seldom to the exclusion of physical remedies.

**Common Disorders and Diseases of Childhood.** By G. F. STILL, LL.D., M.D., F.R.C.P. Fifth edition. Pp. xiv., 1,032. Illustrated. London: Oxford University Press. 1927. Price 30s.—To any medical practitioner who has not yet made the acquaintance of this book we commend the opportunity furnished by the appearance of a new edition. It includes some new chapters, one of which—that on vomiting—is an excellent example of the fascinating way in which Dr. Still sets forth his vast clinical experience. Another—that on erythrœdema—is a monograph in little. We do not know of any book on the diagnosis and treatment of disease that has given us greater pleasure or more instruction.

**A Text-book of Infectious Diseases.** By E. W. GOODALL, O.B.E., M.D., B.S. (Lond.). Third edition. Pp. xvi., 718. 71 illustrations, 26 plates. London: H. K. Lewis & Co. 1927. 30s.—This excellent book, which has been a standard work on Infectious Diseases for many years, opens with an interesting discussion of general principles, including Sydenham's "epidemic constitution." Fever, contagion and infection, disinfection and rashes simulating those of specific fevers, are generally reviewed before special diseases are considered. Anaphylaxis is also adequately discussed. The chapter on "Sore Throat" should be especially valuable to the student, and under "Diphtheria and Scarlet Fever" the Schick test and the Dick and Schultz-Charlton reactions are carefully described. In the bacteriology and pathology of each disease due stress is laid on recent experimental work, whilst the details and statistics of the Influenza pandemic of 1918–1919 make interesting reading. The descriptions are very complete, and even the "common cold" has its allotted place. The section on "Encephalitis Lethargica" is very good. Under "Measles and Rubella" most persons will agree that "the case for the separate existence of a so-called 'Fourth Disease' has not been established." The illustrations of small-pox are excellent, but most of the others serve to emphasise the author's dictum that "skin rashes are best learnt and identified at the bed-side." Regarding Vaccinia, on page 518 details are given of encephalitis arising as a sequel to vaccination, and the rarity of this complication should not affect the use of this safeguard against small-pox. The author does justice to a very big subject, and the book may be confidently recommended to student and practitioner alike.

**Surgical "Don'ts" and "Do's."** By C. H. WHITEFORD, M.R.C.S., L.R.C.P. Second edition. Pp. 68. London: Harrison & Son. 1928. Price 4s.—This small book had originally ten chapters or series of "don'ts" and "do's," and it has now been enlarged to contain fifteen, so that presumably the author has found that his words of wisdom have been appreciated. Many of the dicta only represent well-accepted surgical principles, *e.g.* "Don't perform prostatectomy in the presence of retention of urine." Others give dogmatic advice about doubtful points which would not be generally acceptable, *e.g.* in acute appendicitis "don't forget to insert a drainage tube." Others again give advice about ethical matters which cast rather a reflection on the average practising surgeon. The tract is racy, readable and short.

**Mental Disorders.** By H. J. NORMAN, M.B., B.Ch. (Edin.), D.P.H. (Edin. and Glas.). Pp. xvi., 463. 57 illustrations. Edinburgh: E. & S. Livingstone. 1928. 14s.—This is a handy-sized book for students and practitioners. While not pretending to be an exhaustive treatise on mental diseases, it will well repay study by all interested in this class of cases. It is instructive, concise, and to the point. In discussing the etiology of the various disorders, the author takes a very common-sense view, and does not mistake cause for effect. In the text he also defines the meaning of a large number of recently-coined words, a practice of great advantage to those not well versed in the modern descriptions of the psychoses. His chapters on Dementia Præcox and the Intoxications are particularly good. The notes on general treatment are sound and eminently practical, but he does not discuss the legal position of one who forcibly feeds an *uncertified* patient, and the amount suggested for a single feed, 1½ pints, would appear to be rather excessive in most cases. For very good reasons he prefers the œsophageal to the nasal tube. His suggestion that chloral may be used in from 10 to 40 grain doses should be followed with caution. His clinical examples in life and literature, though they have little bearing on the modern study of mental diseases, are extremely interesting and his criticisms good. The chapter on clinical examples in literature is crisp and amusing, and one would have liked to see it extended. It is to be hoped that the work will have the circulation it deserves, as it should do much to remove some of the prejudices in dealing with the mentally afflicted.

**Chronic Constipation.** By J. ELLIS BARKER. Pp. 503. London: John Murray. 1927. Price 7s. 6d.—We are not told who Mr. Ellis Barker is, beyond the fact that he has written pseudo-medical works dealing with health and cancer. Apparently his chief qualification for writing the present book is that he has been a sufferer from the disease himself. We are not persuaded, however, by the evidence of this book that the patient is the best person to write a fair account of the condition from which he is suffering. It is perhaps hardly necessary to say that the main thesis is that which has been so diligently promulgated by Sir Arbuthnot Lane during recent years, and the book is dedicated to this authority. We are told that constipation causes insanity, rheumatism, cancer—in fact, it would save time if one were to put down a list of those rare diseases which are not caused by constipation. The book does not redeem its violation of all scientific principles by being either racy or readable, and it consists very largely of quotations from medical writers both ancient and modern, many of which are so taken from their text as to be quite unfair and misleading. The attitude of the author towards scientific workers and writers is determined chiefly by their attitude towards his fundamental doctrine. In the main scientists, physicians and the medical profession are condemned for being ignorant and prejudiced. For example, "The unteachable chemists still fill their text-books with worthless information of food values, etc." But where it suits his purpose scientific discovery is greatly belauded, as for example the action of vitamins. It would be much more reasonable if the author left out all appeal to science and contented himself with an appeal for a return to Nature and primitive habits. Daily evacuation of the bowels is spoken of as the "one stool a day fetish," and we are told that the bowels should act three or four times a day. This kind of writing may be admirably fitted for the more exotic popular press, but it is certainly not worthy of a place on the shelves of a medical library.

**The Abdominal Surgery of Children.** By L. E. BARRINGTON-WARD, Ch.M., F.R.C.S. Pp. xiv., 283. Illustrated. London: Oxford University Press. 1928. Price 15s.—This is a book for which there has been a real need. It is concise, and therefore easy for the student to read. It is full of facts, and therefore useful as a reference book after qualification. The text is clearly written and the photographs are really excellent. For hernia operations the author advises confining the operation to removing the sac only. It would have been interesting if, after describing the operation for imperfectly descended testis, he had given us his experience of the after results. The chapter on appendicitis is very complete. The author's experience, in regard to the use of X-rays for this disease, does not conform with that expressed at a recent meeting of the Royal Society of Medicine. The descriptions on pneumococcal and streptococcal peritonitis may be given special praise, but this high standard is well maintained throughout the book.

**The Surgical Treatment of Malignant Disease.** By Sir HOLBURT J. WARING, M.S., F.R.C.S. Pp. xx., 667. 296 illustrations. London: Oxford University Press. 1928. Price 50s.—The author, who has had the unrivalled experience of St. Bartholomew's Hospital to draw upon, gave the Bradshaw Lecture at the Royal College of Surgeons on the Surgical Treatment of Malignant Disease in 1921. This forms the first chapter of the present volume, whilst the remainder is occupied by a systematic account of the different organs as affected by malignant new growths. The work in the main is clinical, but the illustrations give a vast amount of pathological information. As far as possible the late results of treatment are given in all cases, but these are supplied chiefly from the published papers of other surgeons, and not from the impartial "follow-up" records of the hospital, which has only recently instituted this system. The work provides a valuable account of various methods of operation, but it is disappointing in not giving some clear indication as to the results which may be obtained by these operations. For example, in cancer of the tongue the classical operations which involve division or resection of the mandible are clearly described and beautifully illustrated, but there is no guidance as to whether such operations are really worth while. When we look for an account of late results, too, we only find a reference to American figures, which relate that 92 per cent. of cases without glandular involvement are well five years after operation, and 66 per cent. of cases in which only the primary growth was removed were well at a like period, and we feel that this is an optimistic report of a quite misleading character. The author is very guarded in his expressions of opinion about the value of radium and X-rays, and he quotes and illustrates cases in which these have failed, and only admits in a qualified manner that cases are ever cured or alleviated. He mentions the lead treatment, but states that it has only given negative results at St. Bartholomew's Hospital. The number and beauty of the pictures and the great mass of material facts with extensive bibliography all make the work one of outstanding value for purposes of reference.

**Diseases of Nose, Throat and Ear.** By D. MACKENZIE, M.D., F.R.C.S.E. Second edition. Pp. x., 677. 254 illustrations. London: Wm. Heinemann Ltd. 1927. Price 45s.—Since the appearance of the first edition eight years ago, this work has increased until it occupies nearly 800 small quarto pages; it has thus become distinctly a volume for reference rather than for systematic reading. Part of the bulk is due to a style which, while always pleasant, is somewhat discursive; part to inclusion of diseases of the mouth, tongue and thyroid gland, which might well have been omitted from a work on the ear, nose and throat, especially as the treatment is too superficial to be trustworthy. When we have said this, nothing remains but praise for a luxurious and careful exposition of the diseases of these regions, their course and management. Especially valuable are the anatomical sections. The author's own well-known views colour the whole, and impart the personal tone which is so valuable. The illustrations are numerous, excellent and well chosen. For its purpose it is a book we can thoroughly recommend.

**Practical Birth Control.** By ETTIE A. HORNIBROOK. Sixth impression. Pp. xiv., 53. London: Wm. Heinemann Ltd. 1927. Price 3s. 6d.—In this book the authoress sets out the various contraceptive methods, and gives information as to their application and use. She advises the combination of two contraceptives as the only safe method of birth control, and as this is true, the poor woman, who is most in need of this control, is deprived of it on account of the complication of its application and its expense. There is a good chapter on the need for birth control and one on the surgical dilatation of the hymen prior to marriage. Some of the details of hygiene, such as the daily douching of a non-infected vagina, will not receive medical support. However, the book is well worth the attention of doctors and lay women, for whom it is evidently written.

**Post-mortem Appearances.** By J. M. ROSS, M.D. Second edition. Pp. x., 225. London: Oxford University Press. 1928. Price 7s. 6d.—This is an excellent little book, which has already made a name for itself. The first few pages describe the technique of post-mortem examination, including the collection and preservation of material for further investigation. Then follow sections on death from causes other than disease, general metabolic and deficiency diseases, general infections, and diseases of the various organs and systems. An appendix contains useful data on anatomy, ossification centres and eruption of teeth. An extremely useful book for the post-mortem clerk, and a valuable pocket reference for the general practitioner.

**Outlines of Dental Science, II.: Dental Bacteriology.** By R. A. BRODERICK, M.B., L.D.S. Pp. vii., 144. Illustrated. Edinburgh: E. & S. Livingstone. 1926. Price 7s. 6d.—A straightforward account of that part of general medical bacteriology of interest to the dental student. Following a description of the usual sterilising, staining, and cultural methods, the author gives a full account of the more important members of the different groups of bacteria. Additional chapters are devoted to mouth organisms, collection and examination of material, bacteriological tests and immunity. The illustrations are good. The dental student will find here all that is necessary to supplement his lectures and practical work.

**Outlines of Dental Science, VII.: Dental Pathology.** By J. L. D. BUXTON, L.D.S. Pp. 194. Illustrated. Edinburgh: E. & S. Livingstone. 1927. Price 7s. 6d.—This book deals with dental caries and other infections of the mouth, and comprises a chapter on odontomes and tumours of the jaw. The author's intention is to supply both student and practitioner with up-to-date information in a concise manner. In this he has succeeded, and the reader will find not only a complete histological description of the various mouth lesions, but also well-written chapters on signs and symptoms and on treatment.

**Outlines of Dental Science, VIII.: Dental Surgery.** By J. L. D. BUXTON, L.D.S. Pp. 173. Illustrated. Edinburgh: E. & S. Livingstone. 1927. Price 7s. 6d.—The author is to be congratulated on his success in treating so wide a subject so well in such a small volume. Of course, there are many omissions. One would have liked mention of such a common and important condition as hypersensitive dentine and its treatment. It is surprising there are no remarks on local anæsthesia with its many uses in dental surgery. The chapter on extraction is good, as is that also on the lower third molar. The account of dental hemorrhage and its treatment could not be improved on. The chapter on fractured jaws is uneven, and there is no mention of interdental wiring, a very useful method of treatment and one which yields excellent results. The book is well illustrated and excellently produced.

**Materia Medica for Nurses.** By A. M. CRAWFORD, M.D. Pp. viii., 85. London: H. K. Lewis & Co. 1927. Price 3s. 6d.—This book is well written, well printed and contains much that is of interest concerning drugs. The first five chapters give information about the sources of drugs, weights and measures, symbols, preparations and doses. The remainder of the pages give the drugs classified according to the parts of the body they act upon. There is much information most useful to nurses, which should be very helpful to them both for examination purposes and during their nursing career.

